# Target Enzymes Considered for the Treatment of Alzheimer's Disease and Parkinson's Disease

**DOI:** 10.1155/2020/2010728

**Published:** 2020-11-09

**Authors:** Namdoo Kim, Hyuck Jin Lee

**Affiliations:** ^1^Department of Chemistry, Kongju National University, Gongju, Chungcheongnam-do 32588, Republic of Korea; ^2^Department of Chemistry Education, Kongju National University, Gongju, Chungcheongnam-do 32588, Republic of Korea

## Abstract

Various amyloidogenic proteins have been suggested to be involved in the onset and progression of neurodegenerative diseases (ND) such as Alzheimer's disease (AD) and Parkinson's disease (PD). Particularly, the aggregation of misfolded amyloid-*β* and hyperphosphorylated tau and *α*-synuclein are linked to the pathogenesis of AD and PD, respectively. In order to care the diseases, multiple small molecules have been developed to regulate the aggregation pathways of these amyloid proteins. In addition to controlling the aggregation of amyloidogenic proteins, maintaining the levels of the proteins in the brain by amyloid degrading enzymes (ADE; neprilysin (NEP), insulin-degrading enzyme (IDE), asparagine endopeptidase (AEP), and ADAM10) is also essential to cure AD and PD. Therefore, numerous biological molecules and chemical agents have been investigated as either inducer or inhibitor against the levels and activities of ADE. Although the side effect of enhancing the activity of ADE could occur, the removal of amyloidogenic proteins could result in a relatively good strategy to treat AD and PD. Furthermore, since the causes of ND are diverse, various multifunctional (multitarget) chemical agents have been designed to control the actions of multiple risk factors of ND, including amyloidogenic proteins, metal ions, and reactive oxygen species. Many of them, however, were invented without considerations of regulating ADE levels and actions. Incorporation of previously created molecules with the chemical agents handling ADE could be a promising way to treat AD and PD. This review introduces the ADE and molecules capable of modulating the activity and expression of ADE.

## 1. Introduction

With the increase of population, particularly aged people, curing various neurodegenerative diseases (ND), whose symptoms are memory loss and cognitive impairment through the loss of neuronal function, has emerged as a new social and economic issue [1, 2]. Alzheimer's disease (AD) and Parkinson's disease (PD) are the most common cases of ND which could be caused by multiple factors, including (i) the accumulation of misfolded amyloidogenic proteins such as amyloid-*β* (A*β*), tau, and *α*-synuclein (*α*-syn), (ii) cholinergic deficit, (iii) metal ion dyshomeostasis, and (iv) oxidative stress induced by overproduced reactive oxygen species (ROS) [2, 3]. Particularly, the aggregates of the amyloidogenic proteins existed in both intracellular and extracellular regions have been a subject of intensive research topic for understanding of the pathology of AD and PD. Among the protein aggregates, soluble oligomer species have been informed to be the most neurotoxic species [4, 5].

AD is the most common form (ca. 60–80%) of dementia. It has been reported that 47 million people suffered from the disease worldwide, and this number is expected to increase to 75 million by 2030 [6]. The significant hallmarks of AD are (i) the shrinkage of the hippocampus and cortex in the brain, (ii) senile plaques mainly composed of A*β* aggregates, (iii) neurofibrillary tangles (NFT) containing the aggregates of hyperphosphorylated tau (ptau), (iv) miscompartmentalization of metal ions, and (v) oxidative stress [7–9]. Based on various previous studies, among those hallmarks, A*β* has been suggested to be a major risk factor of the onset and progression of AD. Therefore, the production, aggregation, and clearance of A*β* were considered to be related to the pathogenesis of AD, and multiple strategies have been tried to treat AD by (i) inhibiting *β*- and *γ*-secretases which are responsible for the generation of A*β* by cleaving amyloid precursor protein (APP) [10, 11], (ii) modulating A*β* aggregation [2], and (iii) enhancing the removal of A*β* [12–15]. Multiple studies revealed that the late-onset AD has been suggested to be associated with the reduction of A*β* clearance [16]. The age-/pathology-related decrease of the concentration and/or activity of A*β*-degrading enzymes (i.e., neprilysin (NEP) and insulin-degrading enzyme (IDE)) could be the triggers of the onset and progression of AD [17].

In addition to A*β*, ptau has been suggested as another potent major cause of AD [18]. Tau expressed in neurons exhibited a high level in neuronal axons. Hyperphosphorylation of tau makes NFTs that eventually form plaques in the brain. Tau plays important roles for neuronal trafficking and keeping the structure of synapse by stabilizing microtubules and sustaining dendrite structure; however, when the protein is hyperphosphorylated and/or fragmentized, it loses the original functions causing neuronal damages [18, 19]. Recent studies performed with transgenic mice revealed that the neurotoxicity might come from ptau oligomers in the early stage of AD [20].

The second most common form of ND is PD (ca. 6 million people were affected by the disease in 2015) which is characterized by the loss of dopaminergic neurons with the symptoms of shaking, rigidity, and slowness of movement [3, 21–23]. Besides physical disorders, emotional problems also occur including depression and anxiety [23]. One of the major reasons for the onset and progression of PD is the loss of dopaminergic neurons caused by the aggregation of *α*-Syn, forming Lewy bodies, in the brain. Then, the damaged neurons undergo cell death leading to the loss of astrocytes and an increase in the number of microglia, particularly in the substantia nigra [22–24]. Thus, *α*-Syn has been considered as the main target for curing PD [25–27].

*α*-Syn is a soluble 14 kDa protein. It exists at presynaptic terminals as a controller of synaptic vesicle trafficking [25]. The aggregation pathways of misfolded *α*-Syn are similar to those of A*β*: (i) containing lag phase, elongation phase, and steady state and (ii) forming aggregates by interacting through the central hydrophobic region composed of 11 amino acids [28–30]. Unlike monomeric *α*-Syn, its aggregates observed in various spots in cells indicating *α*-Syn species could damage both intracellular and extracellular environments. The oligomeric species have been reported to show neurotoxicity by disrupting the lipid membrane as annular A*β* oligomers [31, 32].

**Figure 1:**
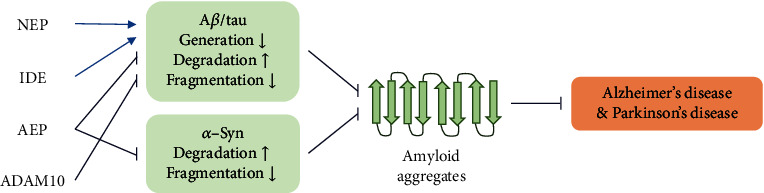
Brief mechanistic scheme of the pathogenesis of AD and PD with an aspect of amyloidogenic proteins and ADE. Regulating the degradation/fragmentation of amyloidogenic proteins by ADE could reduce their aggregation resulting in less risk of onset and/or progression of AD and PD.

To succeed in treating ND, it is necessary to understand the process of generation, aggregation, and degradation of amyloidogenic proteins (i.e., A*β*, tau, and *α*-Syn; Table 1). In order to reduce neurotoxicity caused by the aggregates of these proteins, degrading amyloidogenic proteins could be potent therapeutic methods along with controlling the production or aggregation pathways of amyloidogenic proteins (Figure 1). In this review, we introduce the multiple ADE which are related to multiple ND and their regulators, both inducers and inhibitors toward the expression and/or activity of enzymes.

## 2. Enzymes Related to AD and PD

### 2.1. Neprilysin (NEP)

NEP, known as CALLA or CD10 in the M13 subfamily of type II integral membrane endopeptidases containing 749 amino acids, is predominantly membrane-bound zinc-dependent metallopeptidase with a broad tissue distribution, including the central nervous system, kidney, and vascular endothelium [33–35]. NEP is produced in Golgi and expressed by smooth muscle cells within larger arterioles in the cerebral cortex and leptomeninges in the human brain [35, 36]. It consists of a membrane-spanning helix in the N-terminal region and C-terminal domain with a zinc-binding site, ^583^HEXXH^587^ [37].

In the neutral range of pH, NEP shows the maximum activity [38] and it cleaves the peptide bonds between hydrophobic amino acids, especially phenylalanine and leucine from the small peptides (<50 amino acids) on hydrophobic residues [37, 39]. Hence, the hydrophobic A*β* peptides could be an ideal substrate along with enkephalins, substance P, endothelin, and bradykinin [15, 34]. The active site of NEP is sterically hindered to have high selectivity of substrates. For the monomeric A*β*, multiple peptide bonds formed between (i) E3 and F4, (ii) G9 and Y10, (iii) F19 and F20, (iv) A30 and I31, and (v) G33 and L34 could be cleaved [40]. NEP has been reported to degrade oligomeric A*β* species as well [41].

In the mouse brain, the A*β* levels were shown to increase upon disruption of NEP expression [42]. Lower levels of NEP in cerebrospinal fluid were observed particularly in the prodromal phase and early AD stage [43]. Moreover, inactivation of NEP in the hAPP mouse model caused impaired synaptic plasticity and cognitive performance [42, 44], and high levels of NEP were shown to be colocalized in amyloid plaques [45]. Therefore, NEP has been suggested to be associated with the deposition of A*β* aggregates leading to the onset and progression of AD [46–50]. Based on *in vivo* studies, the upregulation of NEP could be a promising tactic to treat AD not only based on A*β* metabolism but also through other mechanisms (e.g., generating neuropeptide Y fragments) [48–51].

### 2.2. Insulin-Degrading Enzyme (IDE)

IDE is a 113 kDa zinc-dependent metallopeptidase involved in the catabolism of insulin and A*β* [52, 53]. IDE is mostly located in the cytosol; however, it could be found in mitochondria, peroxisomes, plasma membrane, and cerebrospinal fluid as well [54]. It has a ^108^HXXEH^112^ motif for zinc-binding and enzymatic processes. IDE contains 2 similar-sized domains, IDE-N and IDE-C, connected by a loop with 28 amino acids and many hydrogen bonds between two domains keeping the catalytic site closed which is located in IDE-N [55–57]. E111 acts as a base to activate the catalytic water for the hydrolysis of substrates [57]. Thus, IDE-N itself could show the proteolytic activity while the IDE-C domain itself could not have the enzyme activity [58]. The inner side of IDE-N is neutral or negatively charged while the IDE-C domain is positive in charge assisting IDE selects or excludes the substrates based on the charge [56]. Unlike NEP, IDE degrades the substrates containing *β*-structures causing the formation of toxic oligomeric species, subsequently leading to ND, specifically [17, 59].

Since IDE is located in mitochondria and peroxisomes, it could regulate A*β* levels at the intracellular region as well as cover the damages from A*β* aggregates [60–62]. At neutral pH, IDE shows optimal activity to cleave A*β* [63]. IDE is the major enzyme for the removal and cleavage of A*β* in hippocampal lysates, cytoplasm, and cerebrospinal fluid [64, 65]. In addition to degrading A*β* species, the inactive form of IDE could inhibit the fibrillogenesis of A*β* by acting as a chaperone [66].

Although the level of IDE was increased in the hippocampus in the AD-affected brain, the activity of IDE was decreased with aging and at the early stages of AD [63, 67]. Colocalization of A*β* plaques and IDE was observed in the brain suggesting that IDE could be buried in the plaques or oxidized; thus, IDE may lose its amyloid-degrading function [68]. It could lead to a lack of A*β* clearance and protein aggregation resulting in neuronal damages. Also, IDE knockout animals presented the relatively high levels of A*β* [69, 70]. These experimental results proposed that the A*β* cleavage activity of IDE should be maintained to treat ND.

### 2.3. Asparagine Endopeptidase (AEP)

Asparagine endopeptidase (AEP) is a cysteine protease that is also called legumain. Plants legumain was discovered first; then, mammalian legumain was cloned [71, 72]. It cleaves substrates after asparagine residues and sometimes aspartic acid; however, glycosylated asparagines are not cleaved by AEP [73, 74]. It is a member of the peptidase family C13 and has structural homology with other proteases such as caspases and separases [75]. Since legumain is a type I transmembrane protein, it has a signal peptide. Activation of proAEP (56 kDa) requires autocatalytic removal of propeptides at N- and C-terminal (V18-D25 and D324-Y433) regions under acidic condition. Then, the cleavage of these two propeptides and further process produce 46 kDa of active proAEP and 36 kDa of active AEP. Cysteine protease domain (G26-N323) includes catalytic triad (N42-H148-C189) and a few N-glycosylation sites [76, 77]. The optimal pH for enzyme activity is ca. at 5.8; however, it permanently denatures above pH 7.0 [71]. The active site of AEP is blocked by its autoinhibitory C-terminal prodomain which makes the substrate difficult to access.

Moreover, AEP cleaves APP and tau in the brain with *δ*-secretase. Increased AEP level was observed from AD patients' brain samples [78, 79]. Aging is related to an increased AEP level accompanied by an elevated level of fragmented APP. Healthy people have uncleaved APP while AD patients have fragmented APP in the brain [79]. In mouse experiments, general symptoms of emotional problems such as anxiety and depression were reduced after the deletion of AEP knockout. In addition, spatial cognition and learning ability were also improved in AEP knockout mice compared with wild-type mice [80].

Tau and hyperphosphorylated tau can be cleaved by AEP. Cleaved tau (usually tau_1-368_ and tau_1-255_) has strong toxicity in cultured neurons since it favors aggregation [78, 81]. In transgenic mouse experiments, the pH of the brain cortex in tau P301S-expressing mice was lower than the control nontransgenic mice. Likely, AD patients have a slightly acidic environment in the brain than healthy people [82–84]. Low pH might cause activation of AEP in AD patients' brain. AEP also contributes to the splicing process of tau. In AD patients, SRPK2 which is important in pre-mRNA splicing is abnormally activated in tauopathies. Failure of tau exon 10 pre-mRNA splicing regulation results in imbalances in 3R- and 4R-tau. AEP is known to cleave SRPK2 leading to increase kinase activity. Transgenic mice with SRPK2 mutant that cannot be cleaved by AEP showed the elevated synaptic functions and spatial memory while those have truncated SRPK2 underwent 3R- and 4R-tau imbalance leading to accelerate cognitive decline [85, 86]. Tau cleavage by AEP also can be increased by BDNF deprivation. Resulting tau_1-368_ fragment blocks neurotrophic signals on TrkB receptors, eventually leading to cell death [87].

*α*-Syn is also cleaved by AEP, and the fragment of *α*-Syn (cleaved at 103 position) is prone to be aggregated. *α*-Syn cleavage by AEP is age dependent. In the PD animal model and PD patients' brain, aggregated *α*-Syn fragments exhibit high neurotoxicity resulting in neuronal loss and motor impairments. The wild-type and inactive AEP mutant, however, did not cleave *α*-Syn, and little pathogenic effects were observed [22, 23]. Oxidative stress could induce the upregulation of AEP leading to cleave *α*-Syn and neurotoxicity. Based on the previous studies, the cleaved *α*-Syn and activated AEP level showed a close correlation [88, 89]. Therefore, inhibition of AEP activity with synthetic or natural small molecules shed light on the treatment of AD and other ND.

### 2.4. ADAM10

The ADAM family is a zinc-dependent transmembrane and secreted metalloprotease. It usually consists of ca. 750 amino acids as a signal peptide-prodomain-metalloprotease-like domain-disintegrin-like domain-cysteine-rich domain-EGF-like domain-transmembrane domain-cytoplasmic tail. The ADAM family is involved in cell adhesion, proteolytic processing of many receptors, and signaling molecules [90]. ADAM10 is synthesized in the ER and transported to Golgi where it is matured and activated by removal of prodomain. The size of the matured ADAM10, not activated, still containing prodomain, is 68 kDa in Golgi. [91]. ADAM10 forms a dimer, and the C-terminal motif becomes an ordered domain. The zinc-coordinating catalytic core of the ADAM family is HEXGHXXGXXHD, and the active site of ADAM10 in human, rat, and bovine is HEVGHNFGSPHD. Three histidines are responsible for zinc binding, glycine next to phenylalanine allows a turn, and glutamic acid acts as a catalytic residue. Point mutation E384A loses catalytic activity and results in a decrease of soluble APP*α* (sAPP*α*) production which has a neuroprotective function [92]. The disintegrin-like domain is a ligand for integrin binding; however, this domain is not necessary for ADAM10 protease activity [92]. Although the exact mechanism of action has not been known, it is plausible that ADAM10 has a similar catalytic mechanism to other zinc proteases due to the structural similarity of active sites [92].

A*β* can be produced when APP undergoes proteolytic cleavage by *β*- and *γ*-secretases. In contrast, *α*-secretase like ADAM10 does not generate toxic A*β* after cleavage. Instead, the cleavage of APP by ADAM10 produces sAPP*α*. Thus, unlike AEP, inhibition of ADAM10 elevates the neurotoxic A*β* level in the brain and upregulation of ADAM 10 could be a promising way of treatment [93, 94]. Activated ADAM10 proteins were mostly localized on the plasma membrane while proADAM10 still remains in Golgi. This observation indicates there are two pathways to cleave APP: (1) on the cell surface and (2) along the secretory pathway [95].

ADAM10 also plays an important role in regulating synaptic proteins. Neuronal surface ADAM10 undergoes endocytosis by interacting with AP2. In AD patients, the ADAM10/AP2 association was elevated in the hippocampus. ADAM10 activity at the surface decreased upon association with AP2 and endocytosis by long-term potentiation (LTP) in hippocampal neurons [96]. In the ADAM10 knockout mouse model, decreased ADAM10 level resulted in altered network activities in the hippocampus and impaired synaptic function. Consequently, decreased neuromotor abilities and reduced learning abilities were observed. Therefore, reduced ADAM10 might affect the shedding of surface proteins to induce postsynaptic defects [97].

## 3. Regulators toward the Enzymes Related to AD and PD

### 3.1. Regulators of NEP

NEP is one of the major enzymes for A*β* clearance; thus, it has been proposed to be involved in the onset and progression of AD and could be a therapeutic target for caring the disease [15, 98]. Several chemicals have been applied to regulate the activity and expression of NEP. **NNC26-9100** indirectly increases the activity of NEP in cortical tissue [99]. Also, it could decrease APP expression in both cortical and hippocampal tissues causing the reduction of A*β* production. Moreover, **NNC26-9100** inhibits the formation of A*β*_42_ trimers within both extracellular and intracellular cortical fractions. Consequently, the upregulation of NEP expression and activity could lower A*β*_42_ levels and improve memory in an AD transgenic mouse model [99, 100].

In addition, some histone deacetylase (HDAC) inhibitors' potentials as NEP inducers have been investigated [101, 102]. **Valproic acid** could raise the expression and activity of NEP in SHSY-5Y cells as well as the cortex and hippocampus of rats [101, 102]. It could recover cognitive deficits caused by hypoxia in rats [102]. Another HDAC inhibitor, Trichostatin A also presented its ability to enhance the expression of NEP in SHSY-5Y cells [101]. **Imatinib**, known as Gleevec, an anticancer agent, is also proposed to elevate mRNA and protein levels of NEP in multiple cell lines [103]. Recently, **5-hydroxyindoleacetic acid** (**5-HIAA**) also induces the levels of NEP in neuroblastoma cells and *in vivo* [104]. These molecules have potentials to control the A*β* level in the brain and treat AD.

On the other hand, multiple chemical agents such as **phosphoramidon** and **tiorphan** have been reported as NEP inhibitors [40, 105]. They directly interact with NEP at the active site preventing the binding of substrates to NEP [105]. Moreover, chemicals with structural modifications of **phosphoramidon** and **tiorphan**, benzimidazole, and imidazo{4,5-c}pyridine scaffold presented relatively good inhibiting ability against NEP with an IC_50_ value of ca. 0.2-2.0 *μ*M [106]. **MCB3937** is also suggested as NEP inhibitors with a nanomolar range of IC_50_ value [34]. It could interact with the zinc-binding site of NEP, ^583^HEXXH^587^, and ^646^EXXXD^650^, reducing the activity of the enzyme [34]. Another NEP inhibitor is **LCZ696** which is comprised of structural moieties of valsartan and **AHU377**, a prodrug which metabolizes to NEP inhibitor, known as **LBQ657** [107]. The IC_50_ values of valsartan and **LBQ657** against NEP are 2.4 nM and 2.3 nM, respectively [33, 108].

As we introduced above, various molecules have been investigated in order to regulate the activity and expression of NEP. Previously, **NNC26-9100**, HDAC inhibitors (i.e., **valproic acid** and **trichostatin A**), **imatinib**, and **5-HIAA** have been reported as inducers of NEP activity while **phosphoramidon**, **tiorphan**, **MCB3937**, **LCZ696**, **valsartan**, and **LBQ657** were presented as inhibitors of NEP activity. Based on the structures and functions of these molecules, many regulators against NEP activity could be developed.

### 3.2. Controlling Agents toward IDE

The activity of IDE also could be regulated by various substances. Multiple short peptides, including **dynorphin B-9** and **A-17**, have been reported to induce the activity of IDE [109]. Particularly, **dynorphin B-9** increased the A*β* cleavage rate of IDE by ca. 2.5-fold whereas the cleavage rate of other substrates (i.e., insulin) was not affected [109]. **Bradykinin** plays a role as an inducer of IDE activity as well. Upon treatment of **bradykinin**, IDE presents in dimer form which is more active than tetramer form for cleaving various substrates including A*β* [17, 109]. Another short peptide, **somatostatin**, has been shown to increase the cleavage activity of IDE [52]. Two additional exosites have different roles according to the size and binding mode of the substrate to the IDE catalytic cleft: (i) one exosite regulates only the interaction of IDE with larger substrates (i.e., insulin and A*β*_40_) in a differing based on their various modes of binding to the enzyme; (ii) the other exosite is involved in the regulation of enzymatic processing by IDE of all substrates (peptides containing 10-25 amino acids) through the alteration of an open-close equilibrium [52]. **Somatostatin** could bind to both exosites with higher binding affinity and enhance the enzyme activity [52]. In addition to short peptides, **D3**, **D4**, and **D6** developed by Cakir and coworkers presented the possibilities of increasing the activity of IDE toward A*β* degradation through unknown mechanisms [53].

In order to inhibit the activity of IDE, several chemicals have been invented with different modes of action. Firstly, the reported high-affinity IDE inhibitor is **Li-1**. It binds to the catalytic site of IDE with 2 nM as binding affinity and decreases the activity of IDE [110]. Another inhibitor binding to the catalytic cleft to directly interfering the binding of substrates is **BDM44768**, **6bk**, and **NTE-1**. **6bk** and **NTE-1** bind to IDE at a different spot from **Li-1** or **BDM44768**; **6bk** and quinolone-2 moiety of **NTE-1** bind to the pocket located at the outside of the catalytic site, and dipeptide aniline amide analog of **NTE-1** binds to N-terminal anchoring exosite interfering the actions of IDE [111–113]. **BDM41367** suppresses the activity of IDE by binding to both the N-terminal anchoring exosite and catalytic site [114]. **ML345**, containing a thiol group, presented different mechanisms to reduce the cleavage rate of IDE. It interacts with zinc and forms a covalent bond with C819 [115, 116].

To sum up, some biological molecules as short peptide (i.e., **dynorphin B-9** and **A-17**, **bradykinin**, **somatostatin**, **D3**, **D4**, and **D6** (by Cakir and coworkers)) could increase the degrading rate of IDE toward A*β*. Several chemical agents such as **Li-1**, **BDM44768**, **6bk**, **NTE-1**, **BDM41367**, and **ML345**, however, could inhibit the activity of IDE. Previously presented various regulators against IDE could be the first set of library to develop the potent treatment of AD.

### 3.3. Inducers and Inhibitors of AEP

Short peptides such as legumain stabilization and activity modulation (LSAM) domain and *α*_v_*β*_3_ integrin could enhance the activity of AEP. LSAM domain known as the prodomain of AEP blocks substrate binding before activation. This prodomain has a helical structure and two independent peptides. One is an activation peptide (AP, K287 to N323), and the other is a LSAM domain. LSAM domain remains even after AP is cleaved and released from protease at neutral pH *via* electrostatic interaction. AEP without LSAM domain has a lower melting temperature than AEP with LSAM domain [77, 117]. Another short peptide, *α*_v_*β*_3_ integrin, can directly interact with AEP, and after forming a complex, the optimal pH for AEP activity is increased from 5.5 to 6.0. It indicates that *α*_v_*β*_3_ binding could induce conformational stabilization of AEP accompanied by deprotonated C189. *α*_v_*β*_3_ does not directly interact with the AEP active site; however, AEP docks to the *α*_v_*β*_3_ RGD-binding site (allosteric effect) [117]. Based on the immunoanalysis, AEP was mostly found in lysosome and endosome as well as cell surface where *α*_v_*β*_3_ integrin was localized [118]. Naturally occurred polysaccharides with negative charges, glycosaminoglycans (GAGs), could induce the activity of AEP as well. At low pH, proAEP undergoes autocatalytic activation and this process can be accelerated by some GAGs (such as C4S (chondroitin 4-sulfate), C6S (chondroitin 6-sulfate), C4,6S (chondroitin 4,5-sulfate), heparin, and heparin sulfate) with concentration and time dependencies at pH 4.0 [119–121].

On the other hand, various molecules, both short peptides and chemical agents, have been investigated as potent inhibitors against AEP. AENK (Fmoc-Ala-Glu-Asn-Lys-NH_2_) is known to inhibit AEP specifically by blocking the proteolysis of a Dnase inhibitor, SET, both *in vitro* and *in vivo* [74, 122]. AENK selectively inhibits AEP's cleavage activity of *α*-Syn and tau in a dose-dependent manner [22, 78]. Also, SRPK2, which is abnormally activated in tauopathy in AD, is fragmented by AEP and this proteolytic cleavage is inhibited by AENK as well [85]. In addition, Ac-YVAD-CHO (acetyl-Tyr-Val-Ala-Asp-aldehyde) could act as an AEP inhibitor as well as a reversible caspase inhibitor. Ac-ESEN-CHO (acetyl-Glu-Ser-Glu-Asn-aldehyde) is also a specific AEP inhibitor. These two short peptides suppressed AEP activity in the plant leaves [123, 124]. Cystatins consist of two groups of cysteine protease inhibitors (type 1 and type 2) that have conserved a sequence as G9, Q53, V55, and G57. Among cystatins, only cystatin C, E/M, and F (belong to type 2) have inhibitory ability against AEP. Cystatin C inhibits AEP almost completely in a dose-dependent manner. Cystatin C, E/M, and F have AEP inhibitory sites that have chemically similar residues in a loop composed of four amino acids (i.e., SNDM, SNSI, and TNDM for cystatin C, E/M, and F, respectively) [77, 121, 125–130].

Additionally, proteins from fungus could interfere with the actions of AEP. *Clitocybe nebularis* and *Macrolepiota procera* express similar proteins (ca. 20% identical sequence) with 16.8 kDa and 19 kDa, clitocypin and macrocypin, respectively, which could inhibit AEP [131, 132]. Among the five subgroups of macrocypins, only macrocypins 1 and 3 could inhibit AEP [77, 133, 134]. The C-terminal prodomain of AEP itself blocks its active site as autoinhibition, before maturation. Even after maturation, the inhibitory function is still available if the cleaved prodomain is added to the mature AEP [135]. The activated AEP returns back to the inactivated form by autoligation around pH 7.5 because autocatalytic reaction site N323 occurs reversibly [77, 136, 137].

Along with short peptides, various chemical agents also have been revealed as a potent regulator toward AEP. A nonnatural amino acid-based inhibitor **Li-1** was synthesized from aza-peptidyl epoxide. It has ca. nanomolar range of IC_50_ and highly selective against AEP, but not against cathepsins [138]. **Compound 9**, developed by Xu and coworkers, is an aza-Asn epoxide that derived irreversible cysteine protease inhibitor. It showed a concentration-dependent inhibition effect, and the optimal concentration was determined as 1 *μ*M [139, 140].

**R13** is a 7,8-dihydroxyflavone (7,8-DHF)-based prodrug. Although it showed a significant therapeutic effect against AD as a TrkB agonist, 7,8-DHF has two drawbacks: poor oral bioavailability and pharmacokinetic profile. **R13** is the most prominent derivative stemmed from the structure of 7,8-DHF to prevent A*β* deposition in AD mice [141]. Another small molecule, **MV026630**, is an acyloxymethylketone derivative presenting AEP inhibition. It reversibly inhibits AEP and able to be absorbed by living cells [142]. **Compound 11** designed by Zhang and coworkers was selected as an AEP inhibitor by intensive high throughput screening *in vitro* and *in vivo*. **Compound 11** could interact with both the active site and the allosteric site of AEP leading to inhibit the enzyme activity. Upon treatment of **compound 11** to 5xFAD mouse models, both tau and A*β* cleavage were reduced [143].

A thyrotropin-releasing hormone (TRH) analog, **taltirelin**, can be applied orally. **Taltirelin** has 10-100 times enhanced effect on the central nervous system (CNS), and this effect lasts longer than TRH. AD and PD patients have a higher concentration of APP, *α*-Syn, and tau fragments than healthy condition. TRH and its analog, **taltirelin**, lower the phosphorylation level of tau by inhibiting AEP activation. PD mouse research proved that taltirelin can be used to downregulate tau and *α*-Syn-related pathology [144–146]. The AEP activity from bovine kidney and pig was inhibited by *N*-ethylmaleimide, *p*-chloromercuribenzene-sulfonic acid, mercury, and copper as well [71, 147].

In summary, the LSAM domain (i.e., *α*_v_*β*_3_ integrin) and GAGs which are negatively charged glycosaminoglycans could accelerate the activity of AEP. In contrast, (i) tetrapeptide including AENK, Ac-YVAD-CHO, and Ac-ESEN-CHO, (ii) short peptides (i.e., cystatin C, E/M, and F), and (iii) some fungal proteins such as clitocypin and macrocypin could suppress AEP activity. In addition, AEP activity can be self-inhibited by the C-terminal prodomain. Synthetic compounds such as **Li-1**, **compound 9** (by Xu and coworkers), **R13**, **MV026630**, **compound 11** (by Zhang and coworkers), and **taltirelin** act as AEP inhibitors as well. The development of both biological and chemical agents as regulators of AEP could be a key to care AD and/or PD.

### 3.4. Regulating Molecules of ADAM10

Multiple biological molecules such as hormones and transcription factors including XBP-1 (X-box binding protein-1), SOX-2- ((sex-determining region Y-) related high mobility group (HMG) box 2), and PAX2 (paired box gene 2) and chemical agents have been developed to enhance the expression and/or activity of ADAM10. XBP-1 regulates the unfolded protein response pathway. In the transgenic mouse model, ADAM10 expression was 2-fold increased compared to nontransgenic mouse. It was also shown that insulin induces translocation of XBP-1 to the nucleus leading to enhancement of ADAM10 transcription [148]. SOX-2 exhibits a low level in AD patients' brain. SOX-2 is an ADAM10 activator in the nonamyloidogenic processing of *β*APP by ADAM10. HEK293 cells transiently transfected with Sox showed an increased ADAM10 level compared to control with empty plasmid [149]. In addition, a hormone, melatonin, synthesized from tryptophan, could be related to the expression and activity of ADAM10. Its level becomes lower as aging, especially in AD patients. *In vitro* study revealed that melatonin upregulated cleavage of APP by ADAM10 in neuronal and nonneuronal cells. Furthermore, mouse embryonic fibroblasts which have no ADAM10 gene showed reduced melatonin-stimulating function and sAPP*α* secretion [150, 151].

Also, various small molecules have been reported to upregulate the activity and level of ADAM10. **Bryostatin-1**, a macrolide lactone and a PKC activator, enhanced the generation of soluble APP *in vitro* studies even at sub-nM concentration *via* increasing the activity of ADAM10. In the transgenic mouse model, both A*β*_40_ and A*β*_42_ levels in the brain were decreased upon the treatment of **bryostatin-1**. It had several clinical trials for the treatment of cancers as well as AD [152, 153]. **Retinoic acid** and its derivative, **acitretin**, could be an example to elevate the ADAM10 level. 1 *μ*M of **retinoic acid** treatment to SH-SY5Y cells for 4 days results in an increase of ADAM10 mRNA level. **Retinoic acid** could bind to the 302 and 303 nucleotides before the translation initiation site of the ADAM10 gene [154]. Besides, in the experiments with synthetic retinoids, **acitretin** exhibited significant enhancement of nonamyloidogenic processing of APP with an EC_50_ of 1.5 *μ*M [155, 156]. **Am80** is a synthetic retinoid that acts as an agonist for retinoic acid receptors (RAR), RAR*α*, and RAR*β*. **Am80** can increase the ADAM10 transcription level which leads to cleavage of APP. In mouse experiment, mRNA expression and ADAM10 expression were increased upon Am80 administration. **Am80** has been approved for the treatment of acute promyelocytic leukemia in Japan. **Am80** was also under clinical trial for the treatment of AD from 2010 [157, 158]. **Phlogacantholide C** induced ADAM10 transcriptional activity and increased ADAM10 expression level. Consequently, the secretion of ADAM10 that induced APP fragments was elevated [153, 159].

A natural product, **resveratrol**, with a polyphenol framework found in grape skin, peanut, and pomegranates, has been reported to be applied for the treatment of ND to enhance ADAM10 expression indirectly. In mouse experiment, 4 to 5 days of **resveratrol** administration diminished plaque formation [153, 160]. Also, **gemfibrozil** and **etazolate** could induce the levels of ADAM10. Peroxisome proliferator-activated receptor peroxisome proliferator-activated receptor-*α* (PPAR*α*) regulates genes related to fatty acid transport and catabolism and can upregulate ADAM10 expression to increase proteolytic cleavage of APP. **Gemfibrozil**, a PPAR*α* agonist, enhanced ADAM10 expression in isolated mouse hippocampal neurons at 25 *μ*M [93, 153]. **Etazolate** is a GABA_A_ receptor which has an important role in neurotransmission modulator. In rat cortical neuron experiments, concentration-dependent activation of ADAM10 by **etazolate** was observed in the range of 0.2 to 20 *μ*M. *In vivo* studies with guinea pigs revealed that soluble APP level was increased in the brain. In addition, **etazolate** could prevent the neurotoxicity of A*β* on cortical neurons [153, 161].

Along with inducers of ADAM10 expression and activity, various biological molecules and chemicals have been suggested as inhibitors against ADAM10. Firstly, ADAM10 itself exists as an inactive form, zymogen, before the autocatalytic cleavage process. For its activation, removal of ADAM10 prodomain is required and the cleaved prodomain could inhibit the activity of ADAM10 with IC_50_ of 48 nM thorough an unknown mechanism [162, 163]. Also, fish oils, particularly unsaturated fatty acids such as DHA and EPA, which is known to be good for cardiovascular health, could reduce the lease of ADAM10 substrates from endothelial cells. The exact mechanism, however, has not been revealed yet [164, 165].

In addition to biomolecules, multiple chemicals have been examined as potent inhibitors of ADAM10. Low-density lipoprotein receptor-related protein 1 (LRP1) is responsible to transport A*β* across the blood-brain barrier (BBB) resulting in the deposition of A*β* in the brain leading to AD. In activity tests of purified ADAM10 with **GI254023X** and **GW280623**, both compounds inhibited ADAM10 most completely. Long-term treatment of ADAM10 with **GI254023X** in AD mouse increased the A*β* level in the periphery plasma due to reduced LRP1 shedding in the brain [166]. **CID 3117694** is a non-zinc-binding selective inhibitor of ADAM10 with IC_50_ of 6.5 *μ*M. In cell-based assays, **CID 3117694** showed a time-dependent ADAM10 inhibition function. It has similar IC_50_ for several ADAM10 substrate concentrations meaning it is a noncompetitive inhibitor [163].

Hydroxamate derivatives have shown their inhibiting ability against ADAM10 as well. **LT4**, **MN8**, and **CAM29** are hydroxamate derivatives which were developed for selectively inhibiting ADAM10. IC_50_ of **LT4** and **MN8** against ADAM10 are 40 nM and 9.2 nM, respectively [167]. **LT4** (10 *μ*M) also could inhibit ADAM10 sheddase activity carried by ExoV purified from L428 or L540 cells [168]. **CAM29** has an IC_50_ of 20 nM against ADAM10 [169]. Similar to **LT4**, **CAM29** could inhibit ADAM10 sheddase activity carried by ExoV purified from L428 or L540 cells. In addition, CD30 shedding in Hodgkin lymphoma cells was reduced by **LT4** and **CAM29** [168].

Although **INCB3619** has been developed by Incyte Corporation as an ADAM17 inhibitor, it also inhibits that ADAM10 with IC_50_ for ADAM10 and ADAM17 are 0.022 *μ*M and 0.014 *μ*M, respectively [170]. Upon addition to A549 cells, **INCB3619** showed ADAM10 inhibition function leading to deactivation of heregulin and HER3 autocrine signaling [171]. Unlike **INCB3619**, **INCB8765** is an ADAM10 selective inhibitor with 97 nM as IC_50_ against ADAM10 [171].

**Compound 1** and **compound 2** were synthesized and examined as selective ADAM10 inhibitors by Mahasenan and colleagues [172]. **Compound 1**, however, had low potency and selectivity against ADAM10 while **compound 2** has better selectivity toward ADAM10. The phenyl piperidine group in **compound 2** is suitable to occupy the shallow cavity in ADAM10 [172]. Naturally occurring molecules such as rapamycin and **triptolide** also presented inhibiting activity against ADAM10. **Rapamycin** is a widely used drug to prevent rejection in organ transplantation. Recently, **rapamycin** was reported to increase A*β* generation in N2a cells. In addition, the production of sAPP*α* was decreased while *β*-CFT production was increased upon the treatment of **rapamycin**. Two weeks' application of **rapamycin** (3 mg/kg/day) to A*β* overexpressing transgenic mice resulted in elevated A*β* level accompanied by decreased sAPP*α* due to the inhibition of ADAM10 activity [165, 173]. **Triptolide** could be found from a Chinese herb, *Tripterygium wilfordii*. Through affinity chromatography and mass spectrometric analysis, only ca. nanomolar concentration of **triptolide** can downregulate ADAM10 expression in U937 and MCF-7 cells [174].

In short, ADAM10 activity could be elevated by biological molecules such as XBP-1, SOX-2, PAX2, and melatonin. Small molecules such as **bryostatin-1**, **retinoic acid**, **acitretin**, **Am80**, and **phlogacantholide C** and multiple natural products (i.e., **resveratrol**, **gemfibrozil**, and **etazolate**) have been reported as upregulators of ADAM10. On the other hand, biological molecules including ADAM10 prodomain, fish oils (DHA and EPA), and various chemical agents have been known as ADAM10 inhibitors. Synthetic molecules (i.e., **GI254023X**, **GW280623**, **CID 3117694**, **LT4**, **MN8**, **CAM29**, **INCB3619**, **INCB8765**, **compound 1**, and **compound 2** (by Mahasenan and colleagues)) and naturally occurring molecules like **rapamycin** and **triptolide** showed inhibitory effect against ADAM10. With the development of these regulators, AD could be treated by regulating the activity of ADAM10.

## 4. Conclusions

In order to reduce the social and economic burden, therapeutic methods to ND, such as AD and PD, should be developed [1]. Since the presence and aggregation of amyloidogenic proteins, A*β*, tau, and *α*-Syn, could be major causes of the onset and progression of AD and PD [2], it is necessary to control the production, aggregation, and degrading process of the proteins to care the diseases. Based on the published reports we summarized above, regulating the activity/expression of ADE by short peptide and/or chemical agents could be a promising strategy to treat AD and PD; modulation of the expression and/or activity of NEP, IDE, AEP, and ADAM10 presented the clearance of amyloid deposits and improvement of cognitive deficits *in vivo*. The negative effect caused by inducing the activity of ADE could occur; however, the clearance of amyloidogenic proteins may result in a relatively good way to care AD and PD. In addition, it is essential to consider multiple risk factors due to the causes of the diseases that vary. Therefore, recently, various multifunctional (multitarget) small molecules have been invented to control the actions of those amyloidogenic proteins with other risk factors of AD and PD (i.e., metal ions and reactive oxygen species) [2], but most of them do not contain the capability to adjust the actions of ADE. A combination of previously developed molecules with the chemical agents which can upregulate the expression and/or activity of ADE could be the key to the success of treatment of ND, particularly AD and PD.

## Figures and Tables

**Table 1:** The correlation between the activity of ADE and related ND. Different from NEP, IDE, and ADAM10, inhibition of AEP could be a strategy to care AD and PD.

Related neurodegenerative diseases	Enzymes	Results of enhanced enzyme activity	Amyloid aggregation
AD	NEP	A*β* degradation ↑	A*β* aggregation ↓
AD	IDE	A*β* degradation ↑	A*β* aggregation ↓
AD, PD	AEP	Tau/*α*-Syn fragmentation ↑	Tau/*α*-Syn aggregation ↑
AD	ADAM10	A*β* generation ↓	A*β* aggregation ↓
